# Impact of shipping temperature on cell viability and T cell responses to bacterial antigens

**DOI:** 10.12688/wellcomeopenres.18822.1

**Published:** 2023-04-25

**Authors:** Patpong Rongkard, Susanna J. Dunachie, Barbara Kronsteiner

**Affiliations:** 1 Faculty of Tropical Medicine, Mahidol-Oxford Tropical Medicine Research Unit, Mahidol University, Bangkok, 10400, Thailand; 2 Centre for Tropical Medicine and Global Health, Nuffield Department of Medicine, University of Oxford, Oxford, OX3 7LG, UK; 3 Peter Medawar Building for Pathogen Research, University of Oxford, Oxford, OX1 3SY, UK

**Keywords:** IFN-gamma, ELISpot, PBMC, cryopreservation, shipping, storage, melioidosis, Burkholderia pseudomallei

## Abstract

**Background:** Interferon-γ (IFN-γ) secretion by T cells is a key correlate of immune protection against many pathogens including tuberculosis and the neglected tropical disease melioidosis. Clinical studies in tropical regions of immune responses to pathogens and vaccine monitoring studies require the collection of samples in resource-limited rural areas and subsequent shipment to central laboratories for downstream assays and long-term storage.

Here, we studied the impact of two different shipping temperatures on the viability, composition and function of peripheral blood mononuclear cells (PBMC) using multi-colour flow cytometry and IFN-γ enzyme-linked immunospot assay (IFN-γ ELISpot), in order to provide guidance on sample shipment conditions for future clinical studies.

**Methods:** Paired peripheral blood mononuclear cell (PBMC) samples from recovered melioidosis patients were stored in liquid nitrogen (-196°C) and then shipped from Bangkok, Thailand to Oxford, UK at either -80°C (dry ice) or -196°C (dry shipper). After thawing, cell viability and composition were assessed by flow cytometry and antigen specific responses to *Burkholderia pseudomallei* (BP) were measured using IFN-γ ELISpot.

**Results:** We observed modest lowering of viability in the majority of samples and a reduction in IFN-γ responses to BP which correlated to a decrease of monocytes and natural killer cells in samples shipped at -80°C compared to -196°C. Despite being lower in magnitude antigen-specific responses remained detectable in the majority of samples.

**Conclusions:** Here we demonstrate that shipment of cryopreserved PBMC at -196°C has a benefit on cell viability, recovery and T cell responses to bacterial antigens, although useful information can still be obtained from samples shipped at -80°C, thus providing important guidance for sample management in future clinical trials.

## Introduction

Understanding the host immune response to infectious diseases, both during acute infection and for the development of memory responses, is important for the improvement of clinical management, diagnosis and vaccine development. Melioidosis, the neglected tropical disease caused by the intracellular Gram-negative bacterium, *Burkholderia pseudomallei* (BP) *,* is prevalent in tropical areas of the world including Southeast Asia and Northern Australia ^1^ and has high case fatality rates reaching up to 40% in Northeast Thailand ^2^. Melioidosis patients who survive develop strong cell-mediated immune responses with the induction of interferon-γ (IFN-γ) secretion from both CD4 and CD8 T lymphocytes ^3, 4^. Thus, monitoring cellular immune responses in melioidosis is important to inform design of vaccines and novel therapeutics as well as monitor their efficacy.

The enzyme-linked immunospot (ELISpot) assay is widely used to quantify cell-mediated immune responses on a single cell level ^5^. Particularly, the *ex-vivo* IFN-γ ELISpot assay is widely used to assess host-immune responses in immunological studies and those responses are used to define correlates of protection in vaccine studies ^6– 8^. Peripheral blood is the most widely used clinical specimen to study human cellular immune responses. Blood components including serum, plasma and peripheral blood mononuclear cells (PBMC) are commonly cryopreserved at -80°C and then transferred to liquid nitrogen (LN) (-196°C) for long term storage. In the case of melioidosis and other tropical diseases, samples are often collected and processed in rural areas and then shipped to central laboratories for downstream processing or storage. Furthermore, international collaborations necessitate the shipment of cryopreserved PBMC between research centers. Information regarding effects of cryopreservation on cellular immune responses in bacterial infection is still limited and the majority of experimental evidence focuses on monitoring clinical studies of HIV and viral infections ^9, 10^.

Therefore, we compared the impact of two shipping temperatures (-80°C and -196°C) on PBMC viability, phenotype and function in the context of melioidosis in order to provide guidance for future clinical trials. 

## Methods

### Ethical approval and recruitment of subjects

The study protocol was approved by the ethics committees of the Faculty of Tropical Medicine, Mahidol University (submission number TMEC 12-014, reference 081/2555) and the Oxford Tropical Research Ethics Committee (OxTREC 64-11). Blood samples were collected from in-patients with confirmed culture-proven melioidosis at Sunpasitthiprasong Hospital, Ubon Ratchathani, Thailand, as previously reported ^4^. All participants gave written informed consent, including for export and storage of their blood samples.

### Antigen preparation

The crude culture filtrate BP antigen for the *ex-vivo* IFN-γ ELISpot assay was prepared from Thai clinical isolate K96243 as described previously ^11^. Briefly, BP K96243 isolate was cultures in a rice medium at 37°C for 14 days. The culture was autoclaved at 121°C for 15 minutes and centrifuged for 30 minutes at 16,000xg. The culture was filtered through a 0.2micron filter and preserved in a final concentration of 0.5% phenol.

### PBMC isolation and storage

PBMC samples from recovered melioidosis patients were isolated from whole blood using density gradient centrifugation (Lymphoprep, STEMCELL technologies, Cat#07851; Eppendorf centrifuge 5810 R) within three hours after blood collection as described previously ^4^. PBMC (5–10 million cells/mL) were cryopreserved in fetal calf serum (FCS, Invitrogen) supplemented with 10% dimethyl sulfoxide (DMSO, Sigma-Aldrich) at -80°C in a freezing container achieving a rate of cooling of -1°C/min. Patient PBMC were then stored in a -80°C freezer at the study site (for a mean of two weeks) before transport on dry ice to the Mahidol Oxford Tropical Medicine Research Unit in Bangkok for long-term storage in a LN tank. For this study, duplicate PBMC samples from 11 study participants were shipped to Oxford by established commercial shipping carriers using two different temperatures: (i) at -80°C on dry-ice, and (ii) at -196°C in a dry shipper. The samples were in transit (= two days) for the same period irrespective of temperature. Upon arrival, PBMC were immediately stored in a LN tank (Extended datafile 1) ^12^ until used in downstream assays as described below.

### PBMC phenotyping by multicolour flow cytometry

PBMC samples were characterized by flow cytometry based on cell surface markers typically expressed on T, B and NK cells as well as monocytes. Cell viability and apoptosis were determined by staining with Live/Dead (LD) Fixable Near-IR Dead Cell Stain Kit (Life technologies, Cat#L10119, dilution 1:1000) and PE Rabbit anti-Active Caspase-3, monoclonal (mAb) Apoptosis Kit (BD Biosciences Cat# 550914, RRID:AB_393957, dilution 1:8), respectively. The following anti-human mAb antibodies (all raised in mice) were used to stain cells and prepared in cell staining buffer (BioLegend Cat# 420201): CD3-BV510 (BioLegend Cat# 300447, RRID:AB_2563467, dilution 1:100), CD19-FITC (BioLegend Cat# 302205, RRID:AB_314235, dilution 1:200), CD56-APC (BioLegend Cat# 318309, RRID:AB_604098, dilution 1:10), and CD14-PerCP (BioLegend Cat# 325631, RRID:AB_2563327, dilution 1:200). Briefly, cells were stained with the antibodies in the presence of FcR blocking reagent (Miltenyi Biotec Cat# 130-059-901, RRID:AB_2892112, dilution 1:10) and cell viability dye for 20 minutes on ice in the dark. Cells were then washed with 1X phosphate-buffered saline (Sigma-Aldrich Cat#P4417-100TAB) and fixed using Cytofix (BD Biosciences Cat#554714) for 20 minutes on ice in the dark. After fixation, cells were permeabilized using Cytoperm (BD Biosciences Cat#554714) for five minutes on ice in the dark. After permeabilization, cells were stained with PE-anti Active Caspase-3 antibody for 20 minutes on ice in the dark. After the staining, cells were washed and resuspended in MACSQuant running buffer (Miltenyi Biotec Cat#130-111-562). Compensation was performed using BD Comp Beads, anti-mouse Igκ (Miltenyi Biotec Cat#130-097-900) as well as ArC reactive compensation beads (Invitrogen, Cat#A10346). Fluorescence Minus One (FMO) controls were included as controls for compensation and to ensure accurate gating. Cells and compensation beads were acquired using the MACSQuant Analyzer 10 (Miltenyi Biotec). Flow cytometry analysis was performed using FlowJo software version 10.2 on Mac OS X (RRID:SCR_008520). Alternatively, floreada.io, an open-source web-based application can be used to analyse flow cytometry data (.fcs files). PBMC were first gated based on forward (FSC-A) and side (SSC-A) scatter followed by single cell gating using FSC-H and FSC-A. Gating on single cells was performed to distinguish early (LD ^-^Casp3 ^+^) and late (LD ^+^Casp3 ^+^) apoptotic cells as well as dead (LD ^+^Casp3 ^-^) and live (LD ^-^Casp3 ^-^) cells. PBMC populations were gated within the single-live cell gate (LD ^-^Casp3 ^-^) based on the expression of the following cell surface markers: T cells (CD3 ^+^CD19 ^-^), B cell (CD3 ^-^CD19 ^+^), NK cells (CD3 ^-^CD19 ^-^CD56 ^+^), and monocytes (CD3 ^-^CD19 ^-^CD56 ^-^CD14 ^+^) (Extended datafile 2) ^12^.

### IFN-γ enzyme-linked immunospot (ELISpot) assay

PBMC were thawed and rested for one hour in R10 (RPMI 1640 supplemented with 10% FCS, 1mM Penicillin/Streptomycin, 2mM L-glutamine) at 37°C in a humidified incubator with 5% CO _2 _(Eppendorf CellXper C170i). Firstly, multiscreen-I 96 well filter plates (Milipore Cat#MAIPS4510) were coated with 10 ug/mL unconjugated anti-human IFN-γ mAb (MABTECH Cat# 3420-3-1000, RRID:AB_907282) diluted in autoclaved 0.05M carbonate bicarbonate buffer (Sigma-Aldrich Cat#C3041-100CAP) overnight at 4°C. After the incubation, the plates were washed twice with R0 media (without FCS supplement) and blocked with R10 media for one hour at room temperature. Secondly, 2 × 10 ^5^ PBMC/well were added in duplicate to antibody coated plates and incubated in the presence of stimulants for 18–20 hours at 37°C, 95% humidity, 5% CO _2_. Cells were stimulated with heat-inactivated soluble antigens derived from BP strain K96243. Purified protein derivative (PPD, Statens Serum Institut, Denmark, Cat#2390) and Staphylococcal enterotoxin B (SEB, Sigma, Cat#54881) served as positive control and R10 medium was used as negative control. After the stimulation, the plates were washed six times with phosphate buffered saline with 0.05% TWEEN 20 (PBS-Tween 0.05%, Sigma-Aldrich, Cat#P3563). The plates were then incubated with 1 ug/mL biotinylated anti-human IFN-γ mAb (MABTECH Cat# 3420-6-1000, RRID:AB_907272) diluted in 1X PBS for two to four hours at room temperature and washed afterwards. The plates were then incubated with 1 ug/mL Streptavidin-ALP conjugate diluted in 1X PBS for one to two hours at room temperature and washed afterwards. Spots were developed for up to 20 minutes using the AP Conjugate Substrate Kit (BioRad, Cat#1706432). Plates were analyzed on the AID EliSpot Reader (Autoimmun Diagnostika GmbH, Germany, Model#ELR08) using the following settings: thresholds [intensity (min=8, max=255); gradient (min=4, max=90); size (min=22, max=5000)], basic algorithm setting (algorithm C; emphasis=small). Results were reported as spot-forming units (SFU) per million PBMC. The unspecific background (mean SFU from negative control wells) was subtracted from experimental readings.

### Statistical analysis

The differences between matched PBMC subjects were compared using two-tailed Wilcoxon matched-pairs signed rank tests, with significance levels indicated on the graphs. The statistical tests were performed using GraphPad Prism (version 7.0b, RRID:SCR_002798). Alternatively, graphical representation and statistical test can be obtained using ggpubr Bioconductor. Spearman’s correlation analysis was performed using the ggpairs function in GGally R package (version 2.1.1, Bioconductor).

## Results

### Higher shipping temperature has a modest negative effect on cell viability and alters cellular composition

**Figure 1.**
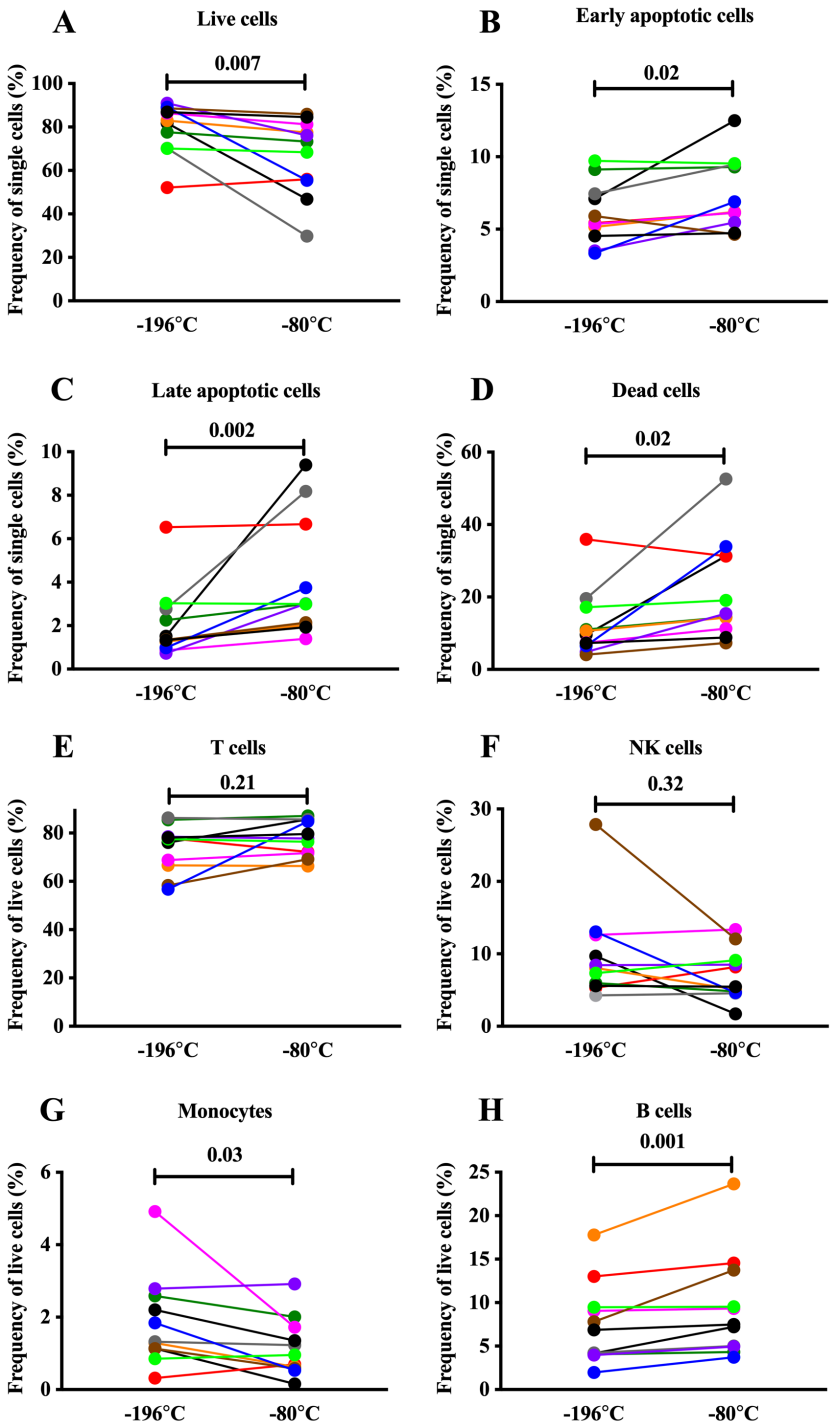
Cell viability and phenotype of PBMC samples following shipment at -196°C and -80°C. Influence of shipping at -196°C and -80°C on cell viability and phenotype of PBMC samples obtained from recovered melioidosis patients one year after infection. ( **A**– **D**) Proportion of live/non-apoptotic, early apoptotic, late apoptotic, and dead cells within single cells. ( **E**– **H**) Proportion of T cells, natural killer cells (NK), monocytes and B cells within single, live cells. Each colored-dot represents one donor. N=11, Wilcoxon paired rank test. Statistical significance levels are indicated on the plot.

A fixable live/dead cell stain and staining for intracellular Caspase 3 activity were used to distinguish between dead, late apoptotic, early apoptotic and live non-apoptotic cells in PBMC from 11 recovered melioidosis patients shipped at two different temperatures. Viability was generally high with a median of 73% and 83% at -80°C and -196°C, respectively. Likewise, seven out of 11 samples showed only minor changes in viability (median: ∆4.5%, range: ∆2–15%) and only three samples displayed a substantial drop (median: ∆35%, range: ∆34–40%) in viability when shipped at -80°C compared to -196°C ( Figure 1A; Underlying Data 1) ^13^. Overall, we observed reduced viability characterised by a significant increase in apoptotic (early and late) and dead cells in samples shipped at -80°C compared to -196°C with high donor variability observed ( Figure 1B–D; Underlying Data 1) ^13^. Phenotypic analysis of the same samples revealed comparable frequencies of T and NK cells between the two shipment temperatures ( Figure 1E, F; Underlying Data 1) ^13^. In contrast, frequencies of monocytes were reduced after shipping at -80°C compared to -196°C (median 0.95% IQR0.58–1.72 vs. 1.32% IQR1.13–2.59, p = 0.03) ( Figure 1G; Underlying Data 1) ^13^. The frequency of B cells was slightly higher in samples shipped at -80°C compared to -196°C (median 7.49% IQR4.95–13.75 vs. 6.87% IQR4.05–9.46, p = 0.001) ( Figure 1H; Underlying Data 1) ^13^.

### Higher sensitivity to cognate antigens in recovered melioidosis samples shipped at lower temperatures

**Figure 2.**
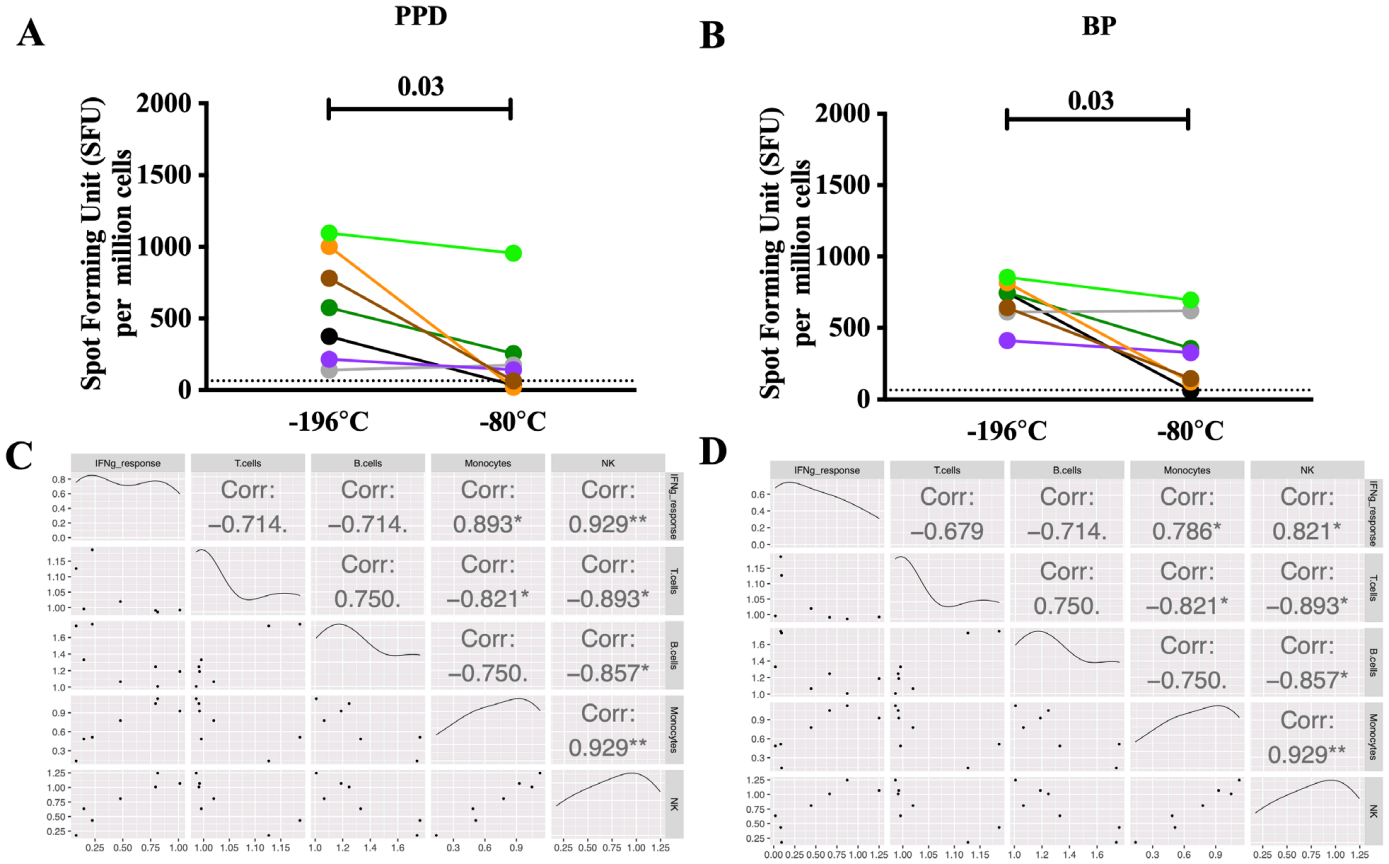
Cellular immune responses in PBMC samples following shipment at -196°C and -80°C. Influence of shipping temperature on cellular immune responses in PBMC samples obtained from recovered melioidosis patients one year after infection. ( **A**, **B**) IFN-γ ELISpot responses to whole cell heat-inactivated *Burkholderia pseudomallei* (HIA-BP) and purified protein derivative (PPD). Each colored-dot represents one donor. N=7, Wilcoxon paired rank test. Statistical significance levels are indicated on the plot. ( **C**, **D**) Spearman’s correlation between cell phenotype (% of live cells) and IFN-γ ELISpot response to BP ( **C**) and PPD ( **D**) respectively. The correlations were calculated using the fold-change difference between the two shipping temperatures (response at -196°C divided by response at -80°C). Each colored-dot represents each donor. Statistical significance levels are indicated on the plot. N=7, * p<0.05, ** p<0.01, *** p<0.001.

Next, we assessed the magnitude of IFN-γ ELISpot responses in seven PBMC samples stimulated with heat inactivated whole cell BP antigen (HIA-BP) and PPD. PBMC shipped at -196°C showed higher IFN-γ ELISpot responses to both HIA-BP (median 745 SFU/10 ^6^ cells, IQR613–855 vs. 328 SFU/10 ^6^ cells IQR123–620, p = 0.03, Figure 2A; Underlying Data 2 and 3) ^13^ and PPD (median 575 SFU/10 ^6^ cells, IQR215–1003 vs. 142 SFU/10 ^6^ cells, IQR35–255, p = 0.03, Figure 2B; Underlying Data 2 and 3) ^13^. In the case of HIA-BP, only one out of seven donors dropped below the detection limit while positive responses against PPD were lost in three out of seven donors when shipped at -80°C compared to -196°C. Importantly, IFN-γ ELISpot responses to both antigen preparations significantly correlate with the frequency of monocytes and NK cells in the sample ( Figure 2C, D; Underlying Data 2 and 3) ^13^.

### Shipping costs

To illustrate the differences in shipping costs for a shipment at -80°C (dry ice) versus -196°C (dry shipper) we compared historic costs from 2016 at the time the samples in this study were shipped with current shipping costs obtained from recent quotes in July 2022 (Extended datafile 3) ^12^. Due to the negative impact of the COVID-19 pandemic on global supply chains and the shipping industry, the costs of sample shipment in 2022 are drastically higher compared to 2016. In our specific example, costs of a dry ice shipment have increased more than three-fold and those for dry shipper transport have risen two-fold. Although the gap between dry ice and dry shipper costs is closing with dry ice being only 2.5-fold cheaper than a dry shipper in 2022 compared to a four-fold difference in 2016, dry ice is still the most cost effective method for international sample shipment (Extended datafile 3) ^12^.

## Discussion

Multi-centre research as well as research involving the collection of specimens in resource limited settings requires regular shipment of samples between laboratories. Clinical samples are precious and often irreplaceable, thus systems have to be in place to ensure the sample integrity is maintained in this process. The ultimate aim when working with cellular material is to preserve cell viability and function. At the same time, other considerations include the availability of certain shipping methods and available budget. In this study, we evaluated the impact of two different shipping temperatures on the viability, cellular composition and antigen specific responses in PBMC from a Thai cohort of recovered melioidosis patients. All samples in our study were maintained under the same storage conditions starting with short term storage at -80°C at the study site followed by a move into LN (-196°C) for archiving at the central laboratory in Bangkok and subsequent intercontinental shipment to Oxford at either -80°C or -196°C. Hence, one batch of samples underwent a temperature change from -196°C to -80°C and back to -196°C while the other batch continuously remained at -196°C.

Here we demonstrate that shipping of PBMC samples at -80°C compared to -196°C results in (1) reduced cell viability, (2) decreased NK cell and monocyte frequency, and (3) diminished IFN-γ responses to HIA-BP and PPD with high donor variability. Despite the reduction in the number of IFN-γ secreting cells, responses to HIA-BP were still detectable (above cut-off) in all but one of the seven samples shipped at -80°C. Overall, our data suggests that storing cells consistently at stable low temperatures is beneficial for cell viability and function.

 The importance of stable deep cryopreservation in preserving antigen-specific immune cells and retaining the greatest sensitivity to the antigens has been highlighted previously in another study showing that constant (-196°C) compared to fluctuating (-196°C to -80°C) storage temperature provided better PBMC recovery with comparable viability and better IFN-γ ELISpot responses against cytomegalovirus, Epstein–Barr virus and influenza (CEF) and CMV ^14^. Another study, in which PBMC samples were shipped at either -196°C or -80°C after overnight freezing at -80°C, showed superior cell viability in PBMC shipped at -196°C, but IFN-γ ELISpot responses to antigens of interest (CEF and CMV) remained unchanged ^15^.

Importantly, our study shows that the reduction in IFN-γ responses to HIA-BP correlates to a loss of both NK cells and monocytes after shipment at -80°C. The HIA-BP preparation used to stimulate PBMC contains a mix of proteins and polysaccharides from the bacterium, thus eliciting not only T cell responses but also triggering activation and cytokine secretion by innate immune cells including NK cells and monocytes ^16^. Our data suggest that the drop in antigen induced IFN-γ secretion measured in the ELISpot assay is mediated by a loss of innate immune cells, which are known to secrete IFN-γ themselves and are crucial for antigen presentation and co-stimulation of T cells.

There are some limitations to this study, which are worth noting. Firstly, cytokine secretion was measured in bulk PBMC, and not on the single cell level using a flow cytometry-based assay. Therefore, it is not possible to determine the impact of shipping temperature on the quality of antigen-specific T cell memory responses including polyfunctionality. Secondly, the specific impact of different shipping temperatures on T cells is unclear, as we used a crude antigen preparation also eliciting bystander responses driven by innate immune cells, thus peptide-specific T cell responses should be evaluated in future studies. Thirdly, our study was not designed to assess the suitability of dry ice shipment for samples previously stored at -80°C. It is worth exploring this further as this might provide a suitable and cost-effective route for sample shipment and at the same time retain quality and function. 

## Conclusions

Our study shows that the ideal shipping temperature for samples previously stored in LN is -196°C. Shipping of samples, which were previously stored in LN, at -80°C can reduce sensitivity of IFN-γ ELISpot responses. Consequently, results obtained from samples shipped on dry ice are not directly comparable to those from samples shipped or continuously stored at -196°C. However, our results suggest that T cell IFN-γ ELISpot responses above detection limit can still be obtained from PBMC kept on dry ice for a number of days, and even though this is less sensitive these responses may be sufficient depending on the research question of interest. This, together with the dramatic increase in shipping costs should be taken into account when designing future trials.

## Consent

Written informed consent for publication of the patients’ details was obtained from the patients.

## Data Availability

Zenodo: Underlying data for ‘Impact of shipping temperature on cell viability and T cell responses to bacterial antigens’, https://www.doi.org/10.5281/zenodo.7737754 ^13^ This project contains the following underlying data: Underlying Data 1- PBMC phenotyping.xlsx Underlying Data 2- IFNg ELISpot and correlation.xlsx Underlying Data 3- IFNg ELISpot (raw images).pdf Figshare: Extended data for ‘Impact of shipping temperature on cell viability and T cell responses to bacterial antigens. https://www.doi.org/10.25446/oxford.21805605.v2 ^12^ This project contains the following extended data: Data file 1: Shipment and storage condition of cryopreserved PBMC samples.png Data file 2: Gating strategy for PBMC phenotyping.png Data file 3: Current and historic shipping costs from Bangkok, Thailand to Oxford, UK.png Data are available under the terms of the Creative Commons Attribution 4.0 International license (CC-BY 4.0)
