# Supplementary material for: Impact of shipping temperature on cell viability and T cell responses to bacterial antigens
Source: Wellcome Open Res. 2023 Apr 25;8:188. [Version 1] doi: 10.12688/wellcomeopenres.18822.1 (PMC11187529; doi:10.12688/wellcomeopenres.18822.1)
Supplement: Peer review history

# Reviewer report: Reviewer response for version 1

**DOI:** 10.21956/wellcomeopenres.20868.r85081

**Published:** 2024-06-19

**Authors:** Sylvia Chieng

**Affiliations:** 1 Universiti Kebangsaan Malaysia, Bangi, Malaysia

This article examines the impact of shipping temperatures on the quality of cryopreserved peripheral blood mononuclear cells (PBMC), which are employed in immune response research against infections such as melioidosis. PBMC samples were shipped at -196°C, which retained cell viability, composition, and T cell responses better than at -80°C, but the latter still gave useful data. These findings provide valuable recommendations for managing sample shipment in clinical investigations done in resource-constrained environments. This article is well-written and sufficient data/methods were provided to allow reproduction.

Is the work clearly and accurately presented and does it cite the current literature?

Yes

If applicable, is the statistical analysis and its interpretation appropriate?

Yes

Are all the source data underlying the results available to ensure full reproducibility?

Yes

Is the study design appropriate and is the work technically sound?

Yes

Are the conclusions drawn adequately supported by the results?

Yes

Are sufficient details of methods and analysis provided to allow replication by others?

Yes

Reviewer Expertise:

Host-pathogen interaction, diagnostic biomarkers, melioidosis

I confirm that I have read this submission and believe that I have an appropriate level of expertise to confirm that it is of an acceptable scientific standard.

# Reviewer report: Reviewer response for version 1

**DOI:** 10.21956/wellcomeopenres.20868.r85085

**Published:** 2024-06-11

**Authors:** Munyaradzi Musvosvi

**Affiliations:** 1 University of Cape Town, Rondebosch, Western Cape, South Africa

In this article, the authors describe a project where they compared the effect of transporting samples at -80°C and -196°C. Peripheral blood mononuclear cells (PBMCs) were isolated from Thai participants who had recovered from melioidosis. The authors found a slight reduction in the viability of samples when they were shipped from Bangkok, Thailand, to Oxford, UK. The authors observed comparable frequencies of T cells and NK cells between the samples shipped at -80°C and -196°C. There was a slight reduction in the frequency of monocytes in samples shipped at -80°C compared to those shipped at -196°C. The authors observed a slight increase in the frequency of B cells in samples shipped at -80°C compared to those shipped at -196°C. Lastly, the authors observed a reduction in the number of IFN-γ producing cells following stimulation with whole-cell heat-inactivated *Burkholderia pseudomallei* or purified protein derivative.

 Overall, the work described in this article was clear, and the study design was appropriate. The conclusions drawn were adequately supported by the data. I have a couple of recommendations that I think would improve the article.

1. In the abstract, the authors mention that “Interferon-γ (IFN-γ) secretion by T cells is a key correlate of immune protection against many pathogens including tuberculosis and the neglected tropical disease melioidosis.” It is true that IFN-γ is a marker that is commonly measured, and it has been shown to be required for protection, but it is not a correlate of immune protection. I recommend that the authors rephrase this statement.
2. I recommend that the authors move the gating strategy shown in Extended Data File 2 to Figure 1. Having the gating strategy next to the summary figures will allow readers immediate access to how the flow cytometry was gated.

Is the work clearly and accurately presented and does it cite the current literature?

Yes

If applicable, is the statistical analysis and its interpretation appropriate?

Yes

Are all the source data underlying the results available to ensure full reproducibility?

Yes

Is the study design appropriate and is the work technically sound?

Yes

Are the conclusions drawn adequately supported by the results?

Yes

Are sufficient details of methods and analysis provided to allow replication by others?

Yes

Reviewer Expertise:

T cell response to M.tb, T cell functional assays, TB vaccine and biomarker development

I confirm that I have read this submission and believe that I have an appropriate level of expertise to confirm that it is of an acceptable scientific standard.
